# Transmission of highly virulent community-associated MRSA ST93 and livestock-associated MRSA ST398 between humans and pigs in Australia

**DOI:** 10.1038/s41598-017-04789-0

**Published:** 2017-07-13

**Authors:** S. Sahibzada, S. Abraham, G. W. Coombs, S. Pang, M. Hernández-Jover, D. Jordan, J. Heller

**Affiliations:** 1 School of Animal and Veterinary Sciences, Charles Sturt University, Wagga Wagga, NSW 2678 Australia; 2 Graham Centre for Agricultural Innovation, Wagga Wagga, Australia; 3 School of Veterinary and Life Sciences, Murdoch University, Murdoch, Australia; 4 PathWest Laboratory Medicine – WA, Fiona Stanley Hospital, Murdoch, Australia; 5 Department of Primary Industries, Wollongbar, NSW 2478 Australia

## Abstract

Pigs have been recognised as a reservoir of livestock associated methicillin-resistant *Staphylococcus aureus* (LA-MRSA) in Europe, Asia and North America. However, little is known about the presence and distribution of MRSA in the Australian pig population and pig industry. This study describes the presence, distribution and molecular characteristics of the human adapted Australian CA-MRSA ST93 isolated from pigs, people, and the environment within a piggery. Isolates were subjected to antibiotic susceptibility testing, DNA microarray, whole genome sequencing, multi locus sequence typing, virulence and resistance gene characterization and phylogenetic analysis. MRSA were isolated from 60% (n = 52) of farm workers where 84% of isolates returned ST93 and the rest ST398. Of the thirty-one pig isolates tested further, an equal number of ST398 and ST93 (15 each) and one as ST30-V were identified. Four of six environmental isolates were identified as ST93 and two as ST398. This study has identified for the first time in Australia the occurrence of CA-MRSA ST93 and LA-MRSA ST398 amongst farm workers, pigs, and the farm environment. Comparative genome analysis indicates that ST398 is likely to have been introduced into Australia from Europe or North America. This study also reports the first linezolid resistant MRSA isolated in Australia.

## Introduction

Methicillin-resistant *Staphylococcus aureus* (MRSA), in addition to being resistant to most beta-lactams, are typically resistant to multiple classes of antibiotics. First reported in a human hospitalised patient in the United Kingdom^1^, MRSA are now frequently isolated worldwide. Although MRSA infections were initially exclusively associated with the hospital setting, a change in epidemiology occurred in the 1990s when infections began to emerge amongst people who had no prior hospital association^2^. These isolates were categorised as community-associated MRSA (CA-MRSA), and based on their accessory genome, staphylococcal cassette chromosome *mec* (SCC*mec*) and epidemiology were easily distinguishable from hospital-associated MRSA (HA-MRSA)^3, 4^. Clinically, CA-MRSA infections are typically skin and soft tissue-related and occur in community members who do not have traditional health care risk factors. In contrast, HA-MRSA are more frequently associated with surgical and systemic infections in the hospital setting^5^. CA-MRSA typically harbors a SCC*mec* IV or V element and additional virulence factors such as Panton-Valentine leukocidin (PVL). Although resistant to beta-lactams, CA-MRSA are generally susceptible to most antimicrobial classes^6^. In contrast, HA-MRSA usually harbour a SCC*mec* I, II, or III element, are PVL negative and are normally resistant to two or more non- beta-lactam antimicrobial classes^7, 8^. However, the distinction between CA-MRSA and HA-MRSA has become blurred with HA- and CA-MRSA crossing environmental boundaries, and in some cases exchanging genetic material^9^. CA-MRSA ST93-IV is the most common CA-MRSA in Australia and it is the second most common strain to cause infection in people, following HA-MRSA ST22-IV^10^.

A third category of MRSA, known as livestock-associated MRSA (LA-MRSA), was reported in the last decade^11^. LA-MRSA emerged independently from CA-MRSA and HA-MRSA with the most frequently isolated LA-MRSA belonging to clonal complex 398 (CC398). CC398 MRSA, first identified in pigs and pig farmers in 2005^12^, has been reported worldwide in a variety of food-animal species^13^ and is the predominant lineage of LA-MRSA in Europe and North America. Although CC398 has been isolated in Asia, LA-MRSA ST9 predominates in pigs in the Asian region^14^. CC398 MRSA has been isolated from up to 50% of pigs in some European countries^15^ and in up to 86% of pig workers^15–17^. While CC398 is frequently reported to be associated with nasal carriage or colonization, infections resulting from this strain in humans, although rare, may also occur^18^. Consequently, CC398 MRSA is recognised as an occupational hazard for people working in the pig industry, including farm workers, veterinarians and abattoir workers^19, 20^.

LA-MRSA transmission and the extent of its ability to persist in pigs and humans are not fully understood. However, it is known that direct animal contact plays an important role in human carriage^19^. A study by Voss and colleagues demonstrated a 760-fold higher MRSA carriage rate among pig farmers compared to the general Dutch population^21^. Duration and intensity of animal contact and the number of MRSA positive animals on a farm have been linked with human CC398 colonization and infection^18, 22^. Furthermore, as CC398 has the ability to survive in the environment, environmental contamination may contribute toward further dissemination. Although occupational exposure to pigs is a risk factor for carriage, compared to CA-MRSA and HA-MRSA, CC398 is not easily transmissible from human to human, and has few virulence-associated genes^23–25^. Furthermore, the prevalence of CC398 has been shown to rapidly decrease during the absence of animal contact^22, 24, 25^. While colonization with CC398 rarely results in human infection^26^, various forms of skin and soft tissue infections, usually minor or localized, have been reported^18^.

In contrast to the knowledge about HA- and CA-MRSA in Australia^27^, little is known about the presence and distribution of MRSA in the Australian pig population and in the Australian pig industry. One cross-sectional study in 2011 examined nasal carriage of MRSA among veterinarians^28^. A single ST398 MRSA^29^ from a pig veterinarian was identified. A second Australian study in 2014 isolated CC398 from less than 1% of 324 pigs sampled from five different commercial pig farms and one feral herd located across Australia^30^.

Despite the limited documentation of LA-MRSA in pigs and pig professionals in Australia, an Australian pig farm was recently identified as being the focus of ongoing MRSA infections amongst its piggery employees over a three-year period. This study aims to describe the presence, distribution and molecular characteristics of MRSA isolated from the people, pigs and environment of the pig production facility where an ongoing outbreak in humans was occurring.

## Methods and Methodology

Approval for the recruitment of human participants into this study was granted by the Charles Sturt University Human Research Ethics Committee (Protocol number 2015/016). Approval for the sample collection from pigs was obtained from the Charles Sturt University Animal Care and Ethics Committee (Protocol number 14/096). All methods were performed in accordance with the relevant guidelines and regulations.

### Sampling

A cross-sectional study was performed at a commercial pig enterprise in Australia to detect the presence or absence of MRSA in people, pigs and the environment. The facility was identified by the farm’s jurisdictional health department due to the recurrent detection of MRSA amongst farm workers with clinical staphylococcal disease. The farm consisted of two sites (designated Farm-A and Farm-B) geographically separated by approximately 40 km. Sampling at each site occurred over a two-day period, with Farm-A visited in May and Farm-B in August 2015.

All farm workers (n = 52) were approached to participate in the study. Participation was voluntary, and signed informed consent was obtained from each participant. A sterile cotton applicator swab (Liquid Amies Elution Swab, Copan ESwab™) was provided and each participant was instructed to rotate the swab in both nostrils for five seconds, before placing the swab in a covered sterile transport tube.

On both farms, pigs were housed in separate sheds based on their age group and class (i.e. dry sows, farrowing, weaners, growers, and finishers). On Farm-A, to optimise sensitivity of the testing procedure, swabs from pigs were collected from the skin caudal and adjacent to the pinnae and from the external nares^31^. With the aim of determining if MRSA was present in the pigs on Farm-A, a previously described pooled sampling method was used: to detect MRSA carriage at a prevalence of at least 2% and assuming 90% test sensitivity with 95% confidence the minimum number of swabs per pool and the minimum number of pools required was calculated to be 10 and 17 respectively, using Epitools online^32^ http://epitools.ausvet.com.au. In addition, in order to obtain estimates of prevalence specific for each shed, six pools (60 swabs) were collected from each shed^33, 34^. From the seven sheds on Farm-A, 420 swabs were collected, providing 42 pools. Animals were randomly selected by farm workers and animals were marked after sampling so re-sampling of the same animals could not occur.

As a large number of MRSA positive pools were found on Farm-A, the sampling procedure was refined for Farm-B. Swabs were collected from the external nares only and samples were processed individually, rather than being pooled. As the prevalence was unknown (and therefore set at 50%) and considering a precision of 5% and 95% confidence, a minimum sample size of 385 swabs was required to determine the prevalence of MRSA in the pigs on this farm. Subsequently, nasal swabs were collected from 408 pigs of different stages of production housed in 13 sheds.

Using established protocols for dust sampling, environmental samples were collected and pooled (five swabs in each pool) from all sheds and effluent collection ponds on both farms^35^. Samples were collected from inside each shed and included walk ways, pens floors, feeders, fences, and walls. Swabs were also collected from the human environment (offices, showers, toilets, and kitchens/sitting rooms).

### Laboratory analysis

All swabs were transported to the microbiology laboratory on ice within 12 hours of collection and immediately refrigerated upon arrival. Swabs were processed within a week of arriving in the laboratory using the isolation method recommended by the European Union Reference Laboratory for AMR (EURL-AMR) for the testing of MRSA in food-producing animals and food samples^36, 37^.

### MRSA isolation

Swabs were pre-enriched in Mueller-Hinton broth (BD™ Difco™) containing 6.5% sodium chloride for 24 hours at 37 °C. Post incubation selective enrichment was performed by transferring one ml of pre-enriched broth into Tryptone Soy Broth (CM0129, Oxoid™) containing 3.5 mg/L cefoxitin and 75 mg/L aztreonam and incubating overnight at 37 °C. Following incubation, a loopful of the selective enriched cultured was inoculated onto chromogenic MRSA agar (CHROMagar™ MRSA) and incubated for 24 hours. As per the manufacturer’s instructions, rose to mauve coloured round colonies were preliminarily identified as MRSA. A single presumed MRSA colony from chromogenic agar was plated onto 5% sheep blood agar and incubated for 24 hours at 37 °C. Cultures were subsequently identified as *S*. *aureus* based on the Gram stain, catalase production and the *S*. *aureus* Protein-A latex agglutination test (staphylase, Oxoid™). *S*. *aureus* ATCC 29213 was used as the control strain.

### Antimicrobial Susceptibility Testing

Antimicrobial disc diffusion susceptibility testing was performed on all 400 *S*. *aureus* identified. Clinical Laboratory Standards Institute (CLSI) disc concentrations and interpretive criteria were used^38^. Antimicrobials tested were: cefoxitin, tetracycline, erythromycin, gentamicin, penicillin, neomycin, ciprofloxacin, chloramphenicol, linezolid, trimethoprim/sulfamethoxazole, vancomycin, teicoplanin, clindamycin, quinupristin-dalfopristin, rifampin, and mupirocin.

Linezolid non-susceptibility was confirmed by minimum inhibitory concentration (MIC) testing using Etest^®^ strips according to the manufacturer’s recommendations (bio Mérieux).

### Molecular characterisation

Molecular testing was performed on 68 MRSA isolates which included the 31 MRSA isolated from humans, and a subset of the pig (n = 31) and environmental (n = 6) MRSA isolates. Pig and environmental MRSA isolates were selected based on their phenotypic antimicrobial susceptibility profiles to include as much phenotypic diversity as possible. The pig isolates were selected to include at least two isolates from each age group on both farms.

### *nuc* and *mecA* characterization

*S*. *aureus* species and methicillin resistance were confirmed by the detection of *nuc* (thermostable extracellular nuclease) and *mecA* (methicillin resistance) genes respectively using multiplex PCR^39^.

### DNA microarray

Isolates were initially characterised using a *S*. *aureus* DNA microarray (Alere Technologies, Jena Germany). Arrays and reagents were obtained from Alere Technologies, Jena Germany. The principle of the assay, related procedures, and a list of targets have been described previously^40^. The microarray was used to detect the presence of virulence and antimicrobial resistance genes. Probes for *mecA*, *ugpQ*, *xylR*, and two probes for *mecR* were used for SCC*mec* typing. The last two probes allowed detection and discrimination for untruncated *mecR* and *mecR*, respectively. Probes for the recombinase genes *ccrA1*, *ccrB1*, *ccrA2*, *ccrB2*, *ccrA3*, *ccrB3*, *ccrA4*, *ccrB4* and *ccrC1*; the fusidic acid resistance marker Q6GD50; and the J region proteins, *dcs*, *plsSCC* and the *kdp*-operon also were included. Ambiguous array results were considered negative.

### Whole genome sequencing (WGS)

Whole genome sequencing was performed using Illumina MiSeq. Genomic DNA libraries were prepared using the Nextera XT kit (Illumina) and sequenced on the 300-bp pair-ended chemistry. Raw sequences reads were *denovo* assembled using CLC genomics workbench (8.5.1).

### Multilocus sequence typing (MLST)

DNA sequences collated at http://saureus.mlst.net/ belonging to 3,044 sequence types (ST) were downloaded in FASTA format and used as the database in the SRST2 pipeline to match the corresponding MLST profiles to the Illumina sequence data^41^.

### *In*-*silico* virulence and resistance gene characterization

The presence of virulence and resistance genes were confirmed using VirulenceFinder, ResFinder tools from Centre for Genomic Epidemiology (CGE) website (www.genomicepidemiology.org) from the FASTA files generated using Illumina sequencing. Additional virulence factors, resistance gene determinants, beyond those detected by VirulenceFinder and ResFinder were determined using CLC Genomics Workbench.

### Comparative Genomics

The MRSA identified in this study were compared to previously published MRSA strains. For ST93 the reference genome JKD6159 (NC_017338) was used as a template and aligned against all contigs to determine SNP locations^42^. The comparative genomic analysis was performed using a comprehensive Australian collection of MRSA ST93 using the 519 SNPs previously reported^43^. The comparative genome analysis of MRSA ST398 was performed by aligning the ST398 genomes sequenced in this study against the reference genome HO 5096 0412 (HE681097)^44^. The reference genome was modified to exclude all phage and intergenic regions. An international collection of MRSA ST398 from a previous study was utilised to perform comparative genomics^23^. SNPs were obtained using Panseq^45^, Maximum parsimony phylogenetic trees, based on the SNPs, were generated for both strains using MEGA (V6.06)^46^.

### Statistical Analysis

All analyses were performed using the R statistical package^47^. Data on inhibition zone size for each drug were converted to dichotomous classification of resistant or susceptible based on inhibition zone diameters recommended in the CLSI documents M100-S24^38^ and VET01-S2^48^. All intermediate resistance isolates were considered as susceptible. Confidence intervals (CI) were calculated at the 95% level of significance.

## Results

### Isolation of MRSA from farm workers, pigs and the environment

MRSA were isolated from 31 (60%) of the 52 farm workers who participated in the study: 10/19 (53%) on Farm-A and 21/33 (64%) on Farm-B.

Forty of the 42 pools of pig samples from Farm-A were MRSA positive. On Farm-B, the prevalence of MRSA-positive pigs was estimated to be 74% (302/408; 95% CI 70–78%).

Twenty five of the 40 pooled environmental samples were MRSA positive (63%; 95% CI 47–76%). MRSA was isolated from 18 of the 20 sheds (90%; 95% CI 70–97%). All seven sheds on Farm-A were positive while 11 were positive on Farm-B. Apart from sheds, the farms’ toilets (2/5), showers (2/4), and kitchens/sitting areas (2/3) were also positive for MRSA. None of the effluent collection ponds or farm-offices grew MRSA.

### Molecular characterization of MRSA

The 68 MRSA selected for molecular characterization were *mecA* and *nuc* gene positive. Initial characterization of the isolates by DNA microarray identified three MRSA strains: ST93 (n = 45), ST398 (n = 22) and ST30 (n = 1). Forty-four of the 45 ST93 isolates carried a SCC*mec* IV element. A SCC*mec* V element was identified in one ST93 and in the ST30 and ST398 isolates.

The 31 human MRSA were identified as either ST93-IV (26 isolates) or ST398-V (5 isolates). Of the 31 pig isolates typed further, 15 were identified as ST398-V, 15 as ST93 (14 harbouring SCC*mec*-type IV and one with SCC*mec*-type V), and one as ST30-V. ST93-IV (4 isolates) and ST398-V (2 isolates) were isolated in the environment.

On Farm-A, all ten MRSA (100%) isolated from the human participants were characterised as ST93. The pig isolates were characterised as ST93 (n = 5) and ST398 (n = 3). ST93 and ST398 were also isolated in the environment.

On Farm-B, 16 of 21 MRSA (76%) isolated from the human participants were characterised as ST93-IV. The remaining five isolates were characterised as ST398-V (24%). Amongst the pig isolates four different MRSAs were characterised: 12 ST398-V, nine ST93-IV, and single isolates of ST93-V and ST30-V. MRSA ST398-V and ST93-IV were also identified in the environment.

### Antimicrobial resistance phenotypes and genotypes

The antimicrobial resistance phenotype and clonal association are described in Table 1. In addition to the β-lactams, in ST93 and ST398 chloramphenicol, clindamycin, erythromycin, neomycin, and tetracycline, resistance was detected. Quinupristin-dalfopristin and linezolid resistance were also detected in ST398. The ST30 isolate was only resistant to the β-lactams and tetracycline.

**Table 1:** Number and proportion of each methicillin-resistant *Staphylococcus aureus* strain resistant to non-β-lactam antimicrobials.

Strain	CHL	CIP	CLI	ERY	GEN	LNZ	MUP	NEO	QDA	RIF	SXT	TEC	TET	VAN
ST398	10 (45.45)	0 (0)	19 (86.36)	18 (81.82)	0 (0)	1 (4.54)	0 (0)	1 (4.54)	8 (36.36)	0 (0)	0 (0)	0 (0)	22 (100)	0 (0)
ST93	45 (100)	0 (0)	37 (82.22)	35 (77.78)	0 (0)	0 (0)	0 (0)	2 (4.44)	0 (0)	0 (0)	0 (0)	0 (0)	12 (26.67)	0 (0)
ST30	0 (0)	0 (0)	0 (0)	0 (0)	0 (0)	0 (0)	0 (0)	0 (0)	0 (0)	0 (0)	0 (0)	0 (0)	1 (100)	0 (0)

CHL (chloramphenicol), CIP (ciprofloxacin), CLI (clindamycin), ERY (erythromycin), GEN (gentamicin), LNZ (linezolid), MUP (mupirocin), NEO (neomycin), QDA (quinupristin-dalfopristin), RIF (rifampin), SXT (trimethoprim/sulfamethoxazole), TEC (teicoplanin), TET (tetracycline), VAN (vancomycin).

All ST93 MRSA carried the chloramphenicol *fexA* resistant determinant (Table 2). Erythromycin (n = 35) and clindamycin (n = 37) resistance was conferred by *ermC*. For the 12 tetracycline resistant isolates 10 carried either *tetL* (9 isolates) or *tetK* (1 isolate).

**Table 2:** Molecular characteristics and phenotypic antimicrobial resistance profile of MRSA isolated from farmworkers, pigs, and environment.

No	Resistance genes	enterotoxin genes	IEC	PVL	Phenotypic antimicrobial Resistance
**Human Isolates** (**31 isolates**)					
**ST398**-**V** (**5 isolates**)					
HT19	*blaZ*, *ermC*, *tetK*, *tetM*, *norA*	−	−	−	BLA, TET, ERY, CLI
H20	*dfrG*, *blaZ*, *ermC*, *vgaA*, *tetK*, *tetM*, *fexA*, *norA*, *lnuB*, *aadE*	−	−	−	BLA, TET, ERY, CHL, CLI, QDA
H21	*blaZ*, *ermC*, *tetK*, *tetM*, *norA*	−	−	−	BLA, TET, ERY, CLI
H23	*ermC*, *tetK*, *tetM*, *norA*	−	−	−	BLA, TET, ERY, CLI
HW-31	*aadD*, *blaZ*, *norA*, *vgaA*, *lnuB*, *cfr*, *fexA*, *tetK*, *tetM*, *dfrG*	−	−	−	BLA, TET, CHL, LNZ, CLI
**ST93**-**IV** (**26 isolates**)					
HT3	*blaZ*, *fexA*, *norA*	ORFCM14	+	−	BLA, CHL
H2	*blaZ*, *ermC*, *fexA*, *norA*	ORFCM14	−	+	BLA, ERY, CHL, CLI
H3	*blaZ*, *ermC*, *fexA*, *norA*	ORFCM14	+	+	BLA, ERY, CHL, CLI
H4	*tetL*, *blaZ*, *ermC*, *aadD*, *fexA*, *norA*	ORFCM14	−	+	BLA, TET, ERY, NEO, CHL, CLI
HT13	*blaZ*, *ermC*, *aadD*, *fexA*, *norA*	ORFCM14	+	−	BLA, TET, ERY, NEO, CHL, CLI
H6	*blaZ*, *fexA*, *norA*	ORFCM14	−	−	BLA, ERY, CHL, CLI
H7	*blaZ*, *ermC*, *fexA*, *norA*	ORFCM14	−	+	BLA, ERY, CHL, CLI
H8	*blaZ*, *ermC*, *fexA*, *norA*	ORFCM14	−	+	BLA, ERY, CHL, CLI
H9	*blaZ*, *ermC*, *fexA*, *norA*	ORFCM14	−	+	BLA, ERY, CHL, CLI
H11	*blaZ*, *ermC*, *fexA*, *norA*	ORFCM14	−	−	BLA, ERY, CHL, CLI
HW-2	*blaZ*, *ermC*, *fexA*, *norA*	ORFCM14	+	+	BLA, ERY, CHL, CLI
H13	*blaZ*, *ermC*, *tetL*, *fexA*, *norA*	ORFCM14	−	+	BLA, TET, ERY, CHL, CLI
H14	*blaZ*, *ermC*, *tetK*, *fexA*, *norA*	ORFCM14	−	+	BLA, TET, ERY, CHL, CLI
H15	*blaZ*, *ermC*, *fexA*, *norA*, *tetL*	ORFCM14	+	+	BLA, TET, ERY, CHL, CLI
H16	*blaZ*, *ermC*, *fexA*, *norA*	ORFCM14	−	+	BLA, ERY, CHL, CLI
H17	*blaZ*, *ermC*, *fexA*, *norA*	seg, egc ORFCM14	−	+	BLA, ERY, CHL, CLI
H18	*blaZ*, *ermC*, *fexA*, *norA*	ORFCM14	−	−	BLA, ERY, CHL, CLI
H19	*blaZ*, *ermC*, *fexA*, *norA*	ORFCM14	−	−	BLA, CHL
HW-15	*blaZ*, *ermC*, *fexA*, *norA*	ORFCM14	+	−	BLA, ERY, CHL, CLI
H24	*blaZ*, *ermC*, *fexA*, *fosB*, *norA*, *tetL*	seg, egc ORFCM14	+	+	BLA, TET, ERY, CHL, CLI
H25	*blaZ*, *ermC*, *fexA*, *norA*	ORFCM14	−	−	BLA, ERY, CHL, CLI
H26	*blaZ*, *ermC*, *fexA*, *norA*	ORFCM14	+	−	BLA, ERY, CHL, CLI
H27	*blaZ*, *ermC*, *fexA*, *norA*	ORFCM14	−	+	BLA, ERY, CHL, CLI
H29	*ermC*, *fexA*, *norA*	ORFCM14	+	+	BLA, ERY, CHL, CLI
HW-24	*blaZ*, *fexA*, *norA*	ORFCM14	+	+	BLA, CHL
H31	*blaZ*, *fexA*, *norA*	ORFCM14	+	−	BLA, ERY, CHL, CLI
**Pig Isolates** (**31 isolates**)					
**ST30**-**V**(**1 isolate**)					
W1Bb-25	*blaZ*, *norA*, *vgaA*, *tetL*, *tetK*	seg, sei, sem, sen, seo, seu, egc	−	−	BLA, TET
**ST398**-**V** (**15 isolates**)					
PTDrAP2	*aadD*, *blaZ*, *norA*, *vgaA*, *ermC*, *lnuB*, *cfr*, *fexA*, *tetK*, *tetM*, *dfrG*	−	−	−	BLA, TET, ERY, CHL, CLI, QDA
PTWeP5	*aadE*, *blaZ*, *norA*, *ermC*, *tetM*, *tetK*, *tetL*	sed	−	−	BLA, TET, ERY, NEO, CLI
PTGrBP1	*aadE*, *norA*, *lnuB*, *cfr*, *vgaA*, *ermC*, *fexA*, *tetM*, *tetK*, *dfrG*	−	−	−	BLA, TET, ERY, CHL, CLI, QDA
W1FPa-4	*blaZ*, *norA*, *ermC*, *tetK*, *tetM*	−	−	−	BLA, TET
W1FSb-2	*blaZ*, *norA*, *tetK*, *tetM*	sed	−	−	BLA, TET
W1FPB-20	*aadE*, *blaZ*, *norA*, *ermC*, *lnuB*, *vgaA*, *fexA*, *tetK*, *tetM*, *dfrG*	−	−	−	BLA, TET, ERY, CHL, CLI, QDA
W1Gr-12	*blaZ*, *norA*, *ermC*, *tetK*, *tetM*	sed	−	−	BLA, TET, ERY, CLI
WweU-9	*aadE*, *blaZ*, *norA*, *ermC*, *lnuB*, *vgaA*, *fexA*, *tetK*, *tetM*, *dfrG*	−	−	−	BLA, TET, CHL
P216	*ermC*, *vgaA*, *tetK*, *tetM*, *fexA*, *norA*, *aadE*, *dfrG*, *lnuB*	−	−	−	BLA, TET, ERY, CHL, CLI, QDA
P221	*blaZ*, *ermC*, *tetK*, *tetM*, *norA*	−	−	−	BLA, TET, ERY, CLI
P223	*blaZ*, *ermC*, *tetK*, *tetM*, *norA*	sea	−	−	BLA, TET, ERY, CLI
P236	*tetK*, *tetM*, *norA*	sea	−	−	BLA, TET, ERY, CLI
P244	*blaZ*, *ermC*, *vgaA*, *tetK*, *tetM*, *fexA*, *norA*, *aadE*, *dfrG*, *lnuB*	−	−	−	BLA, TET, ERY, CHL, CLI, QDA
P257	*blaZ*, *ermC*, *vgaA*, *tetK*, *tetM*, *fexA*, *norA*, *aadE*, *dfrG*, *lnuB*	tst1	−	−	BLA, TET, ERY, CHL, CLI, QDA
P330	*blaZ*, *ermC*, *tetK*, *tetM*, *norA*	−	−	−	BLA, TET, ERY, CLI
**ST93**-**IV** (**14 isolates**)					
PTDrAP4	*blaZ*, *ermC*, *fexA*, *norA*	ORFCM14	−	−	BLA, ERY, CHL, CLI
PTDrBP4	*blaZ*, *ermC*, *fexA*, *norA*	ORFCM14	−	+	BLA, CHL
PTPgP1	*blaZ*, *norA*, *fexA*	sed, seg ORFCM14	−	+	BLA, CHL, CLI
PTGrAP5	*blaZ*, *norA*, *ermC*, *fexA*	ORFCM14	−	+	BLA, CHL
PTF1P3	*blaZ*, *ermC*, *norA*, *fexA*	sed, seh, ORFCM14	−	+	BLA, ERY, CHL, CLI
W1Bb-4	*blaZ*, *norA*, *ermC*, *fexA*	sed, seg, ORFCM14	−	+	BLA, ERY, CHL, CLI
W1FPb-22	*blaZ*, *norA*, *fexA*	ORFCM14	−	−	BLA, CHL
W1Gr-24	*blaZ*, *norA*, *ermC*, *fexA*, *tetL*	sed, ORFCM14	+	+	BLA, TET, CHL, CLI
W1Fi-5	*blaZ*, *ermC*, *fexA*, *norA*	ORFCM14	−	+	BLA, TET, ERY, CHL, CLI
W1Fi-9	*blaZ*, *norA*, *fexA*, *tetL*	sed, ORFCM14	−	+	BLA, TET, CHL
WWeU-6	*ermC*, *fexA*, *tetL*	ORFCM14	+	+	BLA, TET, ERY, CHL, CLI
P271	*blaZ*, *ermC*, *fexA*, *norA*	tst1, ORFCM14	+	−	BLA, ERY, CHL, CLI
P306	*blaZ*, *ermC*, *tetL*, *fexA*, *norA*	ORFCM14	−	+	BLA, TET, ERY, CHL, CLI
P325	*blaZ*, *ermC*, *fexA*, *norA*	seg, egc ORFCM14	−	+	BLA, ERY, CHL, CLI
**ST93**-**V** (**1 isolate**)					
W1Dr-3	*blaZ*, *ermC*, *fexA*, *norA*	seg, ORFCM14	−	+	BLA, ERY, CHL, CLI
**Environmental Isolates** (**6 isolates**)					
**ST93**-**IV** (**4 isolates**)					
ETDrA	*blaZ*, *ermC*, *fexA*	sed, ORF CM14	−	−	BLA, ERY, CHL, CLI
E008	*blaZ*, *ermC*, *fexA*, *norA*	ORF CM14	−	+	BLA, ERY, CHL, CLI
E017	*blaZ*, *fexA*, *norA*	ORF CM14	−	−	BLA, CHL,
E021	*blaZ*, *ermC*, *fexA*, *tetL*, *norA*	seg, egc ORF CM14	−	+	BLA, TET, ERY, CHL, CLI
**ST398**-**V** (**2 isolates**)					
E026	*aadE*, *ermC*, *vga*(*A*), *tet*(*M*), *fexA*, *norA*		−	−	BLA, TET, ERY, CHL, CLI, QDA
ETWeP	*blaZ*, *ermC*, *tetK*, *tetM*, *norA*	sed	−	−	BLA, TET, ERY, CLI

BLA (β-lactam), CHL (chloramphenicol), CLI (clindamycin), ERY (erythromycin), LNZ (linezolid), NEO (neomycin), QDA (quinupristin-dalfopristin), TET (tetracycline).

A large proportion of ST398 were co-resistant to tetracycline, erythromycin, and clindamycin (Table 2). Resistance to erythromycin (n = 18) and clindamycin (n = 19) was conferred by *ermC* (19/22). Tetracycline resistance (n = 22) was conferred by the carriage of the *tetM*, *tetK*, and *tetL* genes. Ten isolates carried the *vgaA* gene which encodes resistance to lincosamides, pleuromutilins and streptogramin A. The lincosamides resistant isolates also carried the *lnuB* gene. Chloramphenicol resistance (10 isolates) was conferred by the *fexA* gene and aminoglycoside resistance (11 isolates) by the *aadD* (2 isolates) and *aadE* (9 isolates) genes. Three ST398 MRSA harboured the *cfr* gene cassette; two from the pig samples and one from a human participant. Only the human isolate was phenotypically linezolid resistant (MIC 32 mg/L). Although the *dfrG* gene was identified in nine isolates, none were phenotypically co-trimoxazole (trimethoprim/sulfamethoxazole) resistant.

The ST30-V isolate was found to carry *tetK*, *tetL* and *vgaA* antimicrobial resistance genes.

### Virulence gene data

The carriage of virulence genes was influenced by the MRSA type (Table 2).

Thirty of the 45 ST93 isolates carried the *luk*-*F*-*PV*/*lukS*-*PV* PVL associated genes: Sixteen of the 26 ST93 human isolates; 12 of the 15 pig isolates; and two of the four environmental isolates. Fourteen ST93 (11 from the human participants and three from pigs) also carried the type B (*sak* + *chp* + *scn*) human immune evasion gene cluster (IEC) genes. All ST93 isolates carried enterotoxin-like ORF *CM14* gene. Enterotoxins *sed* (n = 6), *seg* (n = 7), *seh* (n = 1) and the enterotoxin gene cluster [*egc*] (n = 4) was also detected.

None of the ST398-V isolates carried *lukf*-*PV*/*lukS*-*PV* or IEC genes. Enterotoxins *sea* (n = 2), *sed* (n = 4), and toxic shock syndrome toxin-1 *tst1* (n = 1) genes were detected.

The ST30-V isolate harboured the *egc* complex. The isolate was negative for the PVL and IEC associated genes.

### ST93 phylogeny

**Figure 1:**
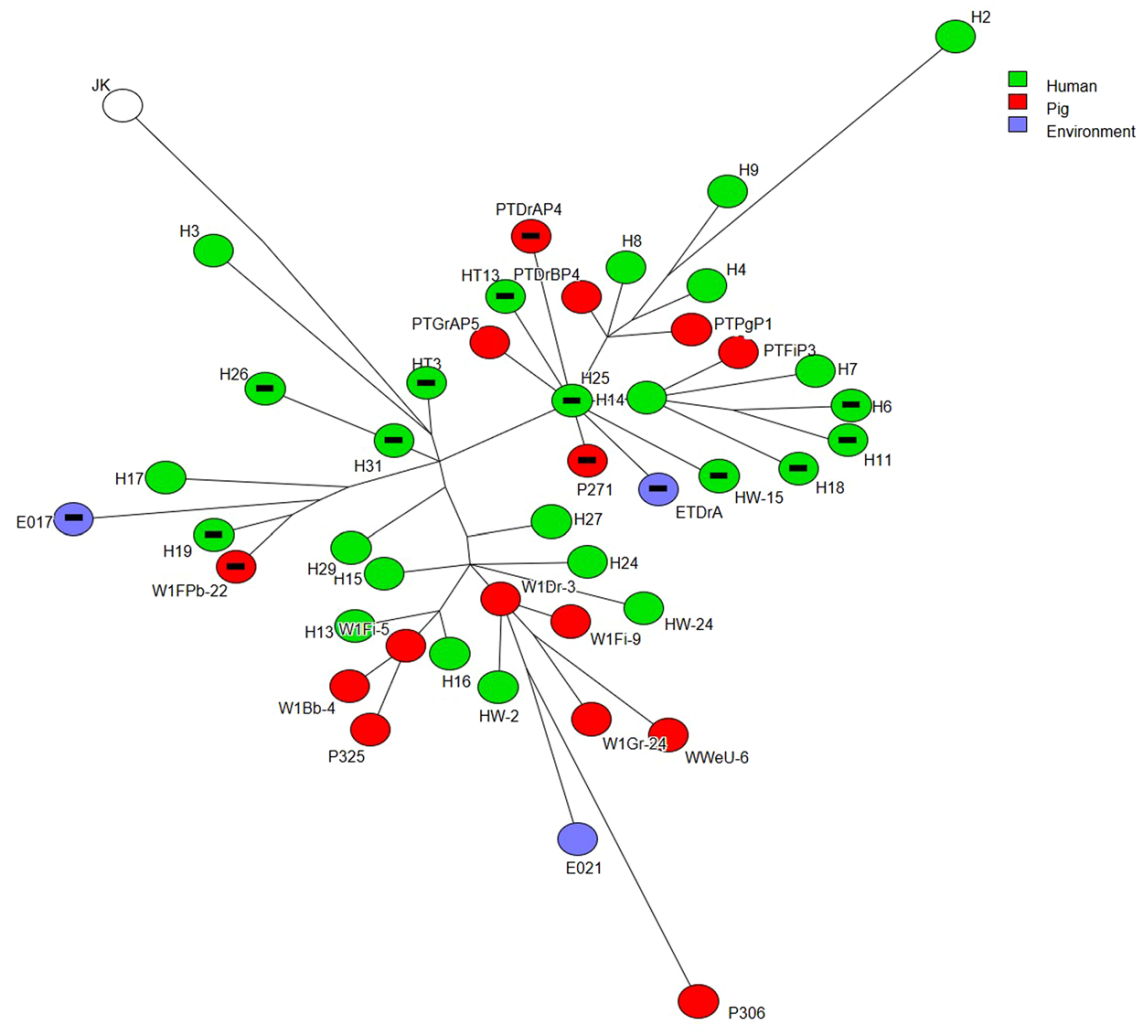
Phylogenetic tree constructed by core genome SNPs of MRSA ST93 isolated from humans, pigs and piggery environment. Each circle represents individual MRSAs. Isolates from humans, pigs, and environment are identified by green, red and purple colours respectively. Isolates that did not carry *pvl* gene are identified by a black bar in the centre.

Between the ST93 MRSA isolates very few SNP mutations were detected. The isolates were closely related to the reference genome JKD6159 (NC_017338) and shared nine common SNP markers. Based on the SNPs the isolates could be classified into three clusters (Fig. 1). There was no relation between the source of the isolates and the cluster.

### ST398 phylogeny

**Figure 2:**
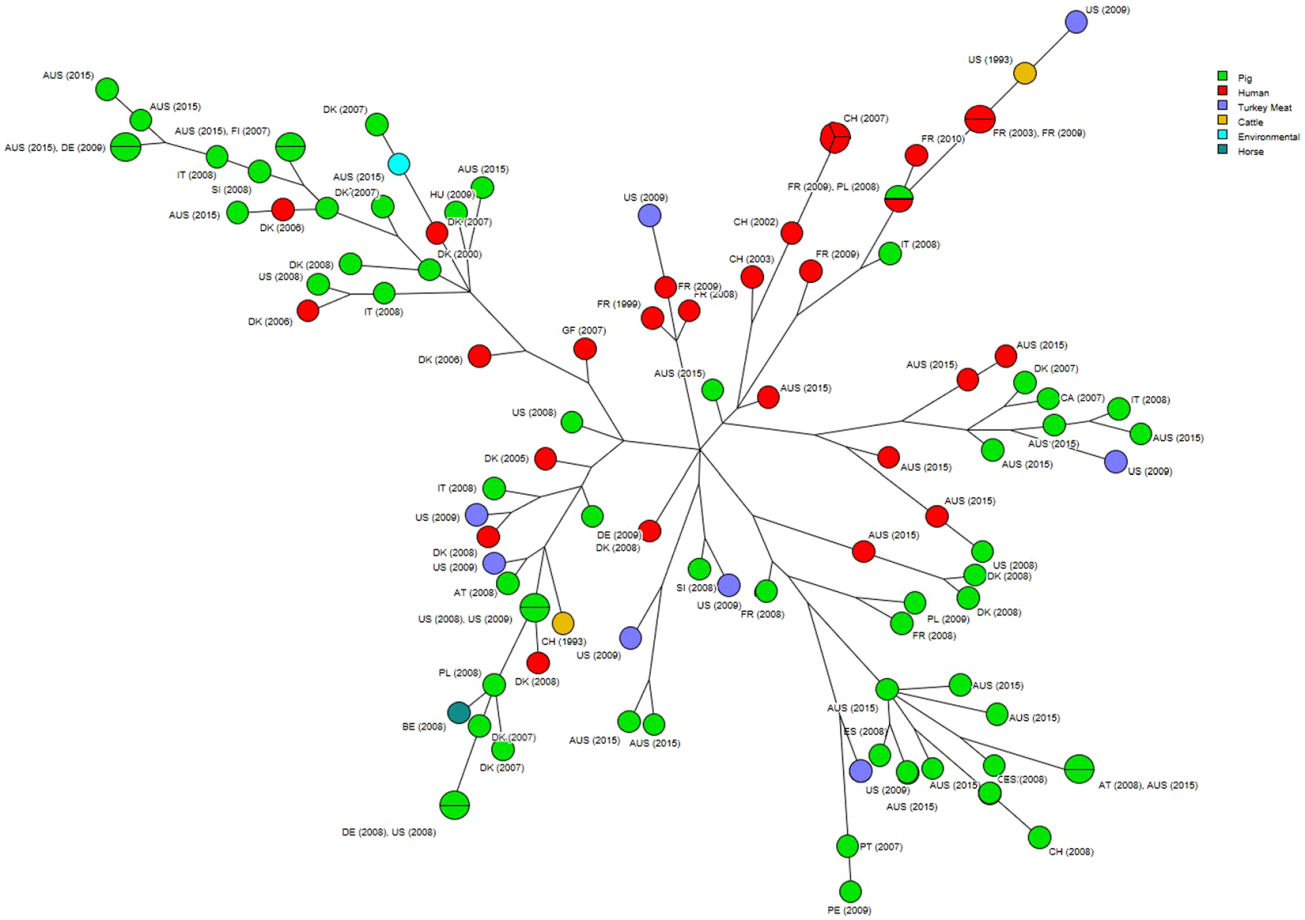
Phylogenetic tree constructed by core genome SNPs of MRSA ST398 isolated from humans, pigs, and piggery environment from this study compared to isolates from different parts of the world. Each circle represents individual MRSAs. Isolates from pigs, humans, turkey meat, cattle, environment and horses identified by green, red, purple, orange, blue and dark green colours respectively. Isolates are coded by the country of origin and year in which they were isolated. Isolates from this study is coded as AUS (2015).

The maximum parsimony tree constructed from ST398 isolates showed three clusters suggesting either multiple introductions of ST398 or an ongoing evolution of the strain. When compared to the international collection of ST398 MRSA genomes, the Australian ST398 isolates from the farmworkers and pigs were similar to the ST398 isolates from Europe and North America (Fig. 2).

## Discussion

This study has investigated the carriage and the molecular characteristics of MRSA isolated from an Australian pig farm spanning two sites (Farm A and B) that had on-going clinical MRSA infections amongst its farm workers.

The study has shown concurrent carriage of MRSA in humans and pigs on both farms with two predominant strains; the Australian CA-MRSA ST93-IV and the international LA-MRSA ST398-V. Furthermore, whole genome sequencing suggests not only has anthropozoonotic and zoonotic transmission occurred, the movement of ST93-IV from human to pig and then back into humans in the presence of the multi-resistant ST398-V has resulted in the acquisition of multiple antimicrobial resistance determinants, such as those encoding for resistance to tetracycline, clindamycin and chloramphenicol, that are not normally found in ST93-IV.

The three major findings arise from this study are as follow: First, we have identified for the first time in Australia the dissemination of CA-MRSA ST93-IV and LA-MRSA ST398-V amongst farm workers, pigs and the farm environment; Second, we have found, using comparative genome analysis, that ST398-V was likely to have been introduced into Australia from Europe or North America and the ST93-IV pig MRSA isolates came from humans with subsequent pig adaptation; and third, to the best of our knowledge we report the first linezolid resistant MRSA isolated in Australia.

ST93-IV, colloquially known as “Queensland CA-MRSA” was first described in the early 2000s in a population of Caucasian humans in Queensland^49^. Typically, ST93-IV has been associated with a range of skin and soft tissue infections in humans only. A singleton by MLST eBURST analysis, ST93-IV has been shown to be highly virulent compared to other well-characterised Australian MRSA, including ST1-IV [2B], ST30-IV [2B] and ST239-III [3B], and the epidemic North American strain, USA300^50^. Since 2014 ST93-IV has become the dominant CA-MRSA across Australia^27^, accounting for approximately 40% of CA-MRSA, 25% of MRSA and 5% of *S*. *aureus* community-onset infections^51^. Consequently, the acquisition of ST93-IV in pigs is likely to have been anthropozoonotic (i.e. human to animal). Although comparative genome analysis showed the porcine and human ST93-IV isolates in our study were closely related to ST93-IV previously characterised in the region (Fig. 1 Supplementary data), the isolates had a lower than expected carriage of the bacteriophage encoded PVL-associated *luk*-*F PV* and *lukS*-*PV* genes. In previous studies upto 100% of ST93-IV have been identified as PVL positive^10, 52^. In contrast, one-third of ST93-IV isolates in our study were PVL negative; 39% of farmworker isolates and 20% of pig isolates. Similarly, a lower than expected carriage of the фSa3 prophage-mediated type B human invasion gene cluster (IEC; *sak*/*scn*/*chp*) was observed. Unlike previous studies, which have shown 100% of ST93-IV harbor a type B IEC, the majority of the pig (80%) and the farmworker (58%) ST93-IV isolates lacked an IEC. The loss of the human associated virulence *lukF*-*PV* and *lukS*-*PV* and *sak*/*scn*/*chp* genes in a large proportion of the ST93-IV isolates suggests the strain has successfully adapted to the porcine host. A similar adaption for *S*. *aureus* moving from the human to porcine host has been reported in CC398^23^.

Contrary to the loss of virulence factors, the ST93-IV identified in our study were phenotypically multi-resistant (including chloramphenicol, clindamycin, erythromycin, neomycin and tetracycline) and harboured multiple resistance genes. Typically, ST93-IV is only beta-lactam resistant with only up to 20% and 2% of isolates resistant to erythromycin and ciprofloxacin respectively^52–54^. It is possible that the observed elevated frequency of resistance to non-beta lactam antimicrobials among the ST93-IV isolates in our study is due to selection within the pig environment, linked to antimicrobial use on the pig farm. The anthropozoonotic transmission of the highly successful CA-MRSA ST93-IV in pigs and the acquisition of additional antimicrobial resistance genes and loss of human associated virulence genes indicate the strain has successfully evolved in the porcine host. This successful evolution may lead to the co-selection, long term maintenance, and further adaptation of ST93-IV in pigs and in the piggery environment^55^.

Prior to our study, ST398-V has only been reported in Australia on two occasions^29, 30^. In our study, 22 isolates were identified as ST398-V (representing 23% of MRSA isolates from farmworkers and also isolated from pigs and the environment). Previous studies have distinguished the livestock-associated *S*. *aureus* ST398 clade from the human clade by the presence of the *tetM* gene and the absence of IEC^56^. As the ST398-V isolated in our study lacked an IEC and a high proportion carried the *tetM* gene it can be assumed the isolates belong to the livestock clade^56^. Comparative phylogenetic analysis suggests the ST398-V was introduced into Australia from Europe or North America^56^. The identification of three ST398-V clusters suggests either multiple introductions into Australia have occurred or the strain has diverged within the farm environment. However, as one of the clusters (cluster 3 versus clusters 1 and 2) differed by more than 34 SNPs over a short period it is more likely that both events have occurred (Fig. 2 Supplementary data).

The importation of CC398 into a country previously free of CC398 and the subsequent transmission into the country’s pig population followed by zoonotic spread is not unique^57^. In Europe, the spread of LA-MRSA between countries is often mediated by animal trading of piglets sold by specialised producers^58^. However, in Australia, the importation of live pigs ceased over 40 years ago. Consequently, it is possible that the introduction of ST398-V into Australia has occurred from pig farmers or pig veterinarians who have had contact with European or North American pig farms, or through the entry of other animal species, such as horses, where the same movement restrictions are not present.

Although *S*. *aureus* does not cause much illness in pigs, CC398 MRSA does cause a wide spectrum of infections in humans, ranging from relatively minor or localised infections to more serious or invasive infections^59^. Furthermore, nosocomial infections and outbreaks have occurred associated with this MRSA. However, similar to other *S*. *aureus*, transmission of CC398 MRSA is primarily mediated by physical contact and overall relatively few cases of CC398 MRSA have occurred in people who are not directly involved in livestock production. The low prevalence of CC398 MRSA in people not associated with pig farming is probably due to the low transmissibility of the organism^60^. Consequently, the clinical significance of CC398 MRSA within the community is thought to be low. However, the emergence of ST398 in the Australian pig industry is a public health concern for those working within the industry. Although ST398 MRSA is less virulent and less transmissible compared to other *S*. *aureus*, the organism exhibits co-resistance to many non β-lactam antimicrobials used in medical and veterinary practice including macrolides, tetracycline, trimethoprim, gentamicin, ciprofloxacin, trimethoprim-sulfamethoxazole, lincosamides and streptogramin B^57, 59^. Although ST398 remains susceptible to the glycopeptides, daptomycin, tigecyclines, fusidic acid and rifampicin, isolated cases of linezolid resistance have been reported^57^. In ST398 MRSA, linezolid resistance is due to the acquisition of a plasmid harbouring the *cfr* gene which in addition to linezolid resistance mediates resistance to lincosamides, fenicols and pleuromutilins^61^. The selective pressure in favour of the spread of the *cfr* containing plasmid may be influenced by the use of linezolid in human medicine and florfenicol and tiamulin in veterinary medicine. The linezolid resistance identified in a ST398 MRSA isolated from a human in this study is the first instance of linezolid resistance in MRSA to be reported in Australia.

Although multidrug resistance may compromise the treatment of ST398 MRSA infections, the reservoir of resistance genes harboured by ST398 MRSA is more concerning from a clinical standpoint. This concern was recently highlighted by Brennan *et al*. following the introduction of CC398 into the Republic of Ireland’s pig industry who suggested that the reservoir of resistance genes harboured by the less virulent and less transmissible CC398 MRSA could potentially spread to other animal and human strains^62^. Unfortunately, this may have occurred in Australia. It appears from our study the movement of the highly virulent and highly transmissible ST93-IV CA-MRSA from humans to pigs and the importation of ST398-V LA-MRSA into the Australian pig farms has resulted in the emergence of a multi-resistant PVL-positive ST93-IV strain which continues to harbour a type B IEC. However, a comprehensive molecular comparison of the antimicrobial resistance genes and the associated mobile genetic elements found in the ST93-IV and ST398-V is required. Furthermore, the fitness cost to ST93-IV following the acquisition of the additional genetic material will need to be determined in order to assess the human health risk, if any, to those animals and humans not exposed to the particular pig herds investigated in this study.

## Conclusion

In conclusion, our study has demonstrated for the first time the widespread occurrence of the highly virulent Australian ST93-IV CA-MRSA and the international ST398-V LA-MRSA strains in an Australian piggery. Furthermore, the evidence suggests that anthropozoonotic and zoonotic transmission may have occurred. The presence of multiple antimicrobial resistance determinants that are not usually found in ST93-IV could be a consequence of the transmission of ST93-IV from humans to pigs and then back into humans in the presence of the multi-resistant ST398-V. The outcomes of this study indicate that in Australia, and probably elsewhere, greater scrutiny of staphylococcal infections in humans and animals is warranted in the form of coordinated investigation of outbreaks to identify the factors predisposing each host species to infection and emergence of virulent pathogens with novel resistance traits.

## Electronic supplementary material

Supplementary Information
