# Supplementary material for: Transmission of highly virulent community-associated MRSA ST93 and livestock-associated MRSA ST398 between humans and pigs in Australia
Source: Sci Rep. 2017 Jul 13;7:5273. doi: 10.1038/s41598-017-04789-0 (PMC5509732; doi:10.1038/s41598-017-04789-0)
Supplement: Supplementary file 1 — Supplementary Information [file 41598_2017_4789_MOESM1_ESM.pdf]

Transmission of highly virulent community-associated MRSA ST93 and livestock-associated MRSA ST398 between humans and pigs in Australia

S. Shafiullah[A,B], *S. Abraham*[C], *G.W. Coombs*[C,D] *S. Pang,*[C,D], *M. Hernández-Jover*[A,B], *D. Jordan*[E], *J. Heller*[A,B*]

[A] School of Animal and Veterinary Sciences, Charles Sturt University, Wagga Wagga, NSW 2678, Australia.

[B]Graham Centre for Agricultural Innovation, Australia.

[C]School of Veterinary and Life Sciences, Murdoch University, Murdoch, Australia

[D]PathWest Laboratory Medicine – WA, Fiona Stanley Hospital, Murdoch, Australia

[E]Department of Primary Industries, Wollongbar, NSW 2478.

Supplementary data.

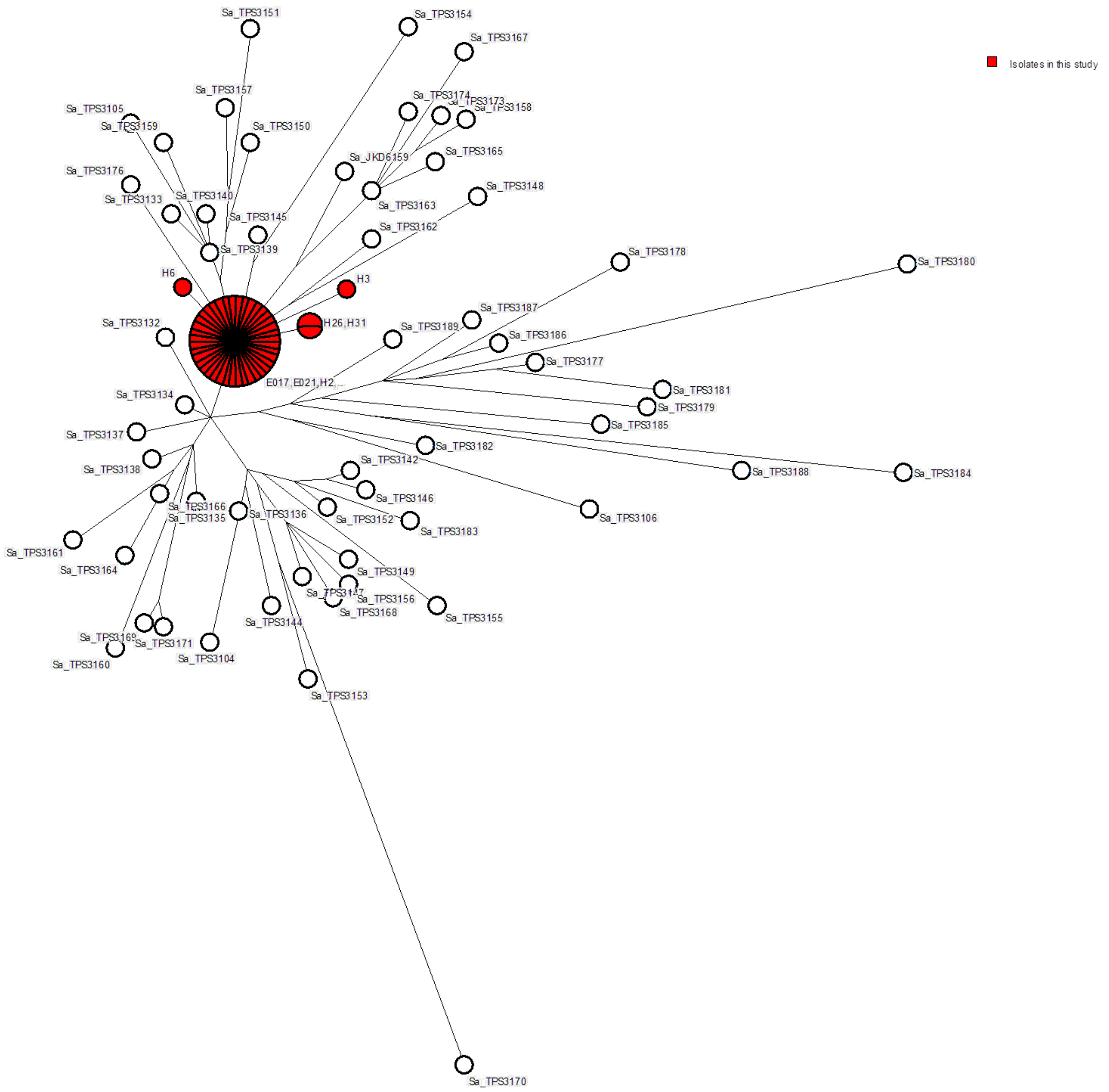

Fig. S 1: supplementary data: Phylogenetic tree constructed by core genome SNPs of MRSA ST93 isolated from humans, pigs, and piggery environment from this study compared to isolates from different parts of Australia. Isolates form this study is represented by a red circle.

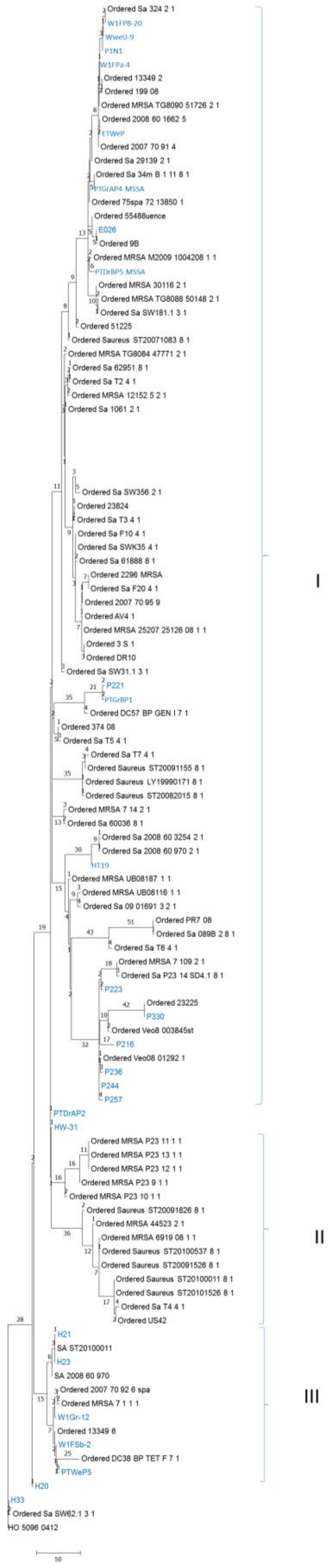

Fig. S 2: supplementary data: Phylogenetic tree constructed by core genome SNPs of Australian MRSA ST398 isolated from humans, pigs and piggery(blue) and compared to international isolates. Isolates are divided into 3 clusters designated as I, II and III
